# Severe acute kidney injury after near-drowning in a healthy adult: A case report

**DOI:** 10.1097/MD.0000000000046402

**Published:** 2026-01-02

**Authors:** Jihao Xu, Bin Zhu

**Affiliations:** a Department of Clinical Medicine, Hangzhou Normal University, Hangzhou, Zhejiang, China; b Urology & Nephrology Center, Department of Nephrology, Zhejiang Provincial People’s Hospital (Affiliated People’s Hospital, Hangzhou Medical College), Hangzhou, Zhejiang, China.

**Keywords:** acute kidney injury, drowning, ischemia-reperfusion, renal biopsy

## Abstract

**Rationale:**

Acute kidney injury (AKI) is an uncommon but serious complication of near-drowning, and its underlying mechanisms remain poorly defined. Existing evidence suggests a multifactorial process, yet reports with integrated clinical and pathological evaluation are scarce.

**Patient concerns:**

A previously healthy man in his thirties presented with progressive oliguria and elevated serum creatinine 2 days after a freshwater near-drowning incident.

**Diagnoses:**

AKI secondary to near-drowning.

**Interventions:**

The patient received individualized fluid management, empirical antibiotics, and intermittent hemodialysis. A renal biopsy was performed to clarify the underlying mechanism.

**Outcomes:**

Renal histology confirmed acute tubular injury. The patient underwent 7 dialysis sessions. Urine output and renal function gradually recovered, and he was discharged on hospital day 11. Follow-up after 1 month showed complete normalization of renal function.

**Lessons:**

AKI following near-drowning may be multifactorial, with ischemia-reperfusion injury being the primary contributor. In ambiguous cases, renal biopsy can provide valuable histological evidence to support diagnosis and guide therapy. Early recognition and tailored supportive strategies are essential for renal recovery.

## 1. Introduction

Drowning is a major cause of unintentional injury-related death worldwide, particularly in low- and middle-income countries, as reported by the World Health Organization.^[1,2]^ In survivors, in addition to life-threatening respiratory failure, multiorgan dysfunction may occur, including acute kidney injury (AKI). AKI is characterized by a sudden decline in renal function, leading to the accumulation of nitrogenous waste (e.g., creatinine, urea) and impaired toxin clearance.^[3]^ AKI occurs in a substantial proportion of drowning survivors, with Gorelik et al reporting an incidence of 43%, including 18% with stage 2 to 3 disease.^[4]^ When present, it can be clinically severe and is linked to poor outcomes if not promptly treated. The pathophysiology is multifactorial, involving ischemia-reperfusion injury, rhabdomyolysis, hypovolemia, and nephrotoxic exposure.^[5]^ Notably, few case reports have described post-drowning AKI confirmed by renal biopsy, especially those with complete renal recovery. Given the potential severity and diagnostic challenges, early recognition and timely supportive therapy are critical for improving patient prognosis. We report a biopsy-proven case of severe AKI requiring renal replacement therapy in a previously healthy 35-year-old man following freshwater drowning, which provides crucial insights into the underlying pathophysiology and the role of individualized management.

## 2. Case report

A 35-year-old previously healthy man presented with a 2-day history of oliguria following a freshwater near-drowning incident during recreational fishing in the Fuchun River. He had accidentally fallen into the river and struggled in the water for nearly an hour before managing to reach the shore unaided. He reported no history of trauma, crush injury, or prior renal disease. At the referring hospital, initial tests revealed microscopic hematuria (2+), heavy proteinuria (3+), and a markedly elevated serum creatinine level of 487 μmol/L. Abdominal non-contrast computed tomography showed bilateral renal enlargement with perirenal and periureteric stranding, while chest computed tomography demonstrated ventilation-perfusion mismatch and mild chronic inflammation in the right middle lobe. Due to progressive renal dysfunction (creatinine 626.1 μmol/L), he was referred to our institution for further evaluation and management.

Upon admission, the patient was alert and hemodynamically stable, although his blood pressure was elevated at 151/102 mm Hg. Fine crackles were noted at both lung bases. Laboratory tests revealed leukocytosis (white blood cells 10.23 × 10⁹/L, ANC 8.78 × 10⁹/L), elevated C-reactive protein (40.2 mg/L), and mild anemia (Hb 126 g/L). Urinalysis confirmed heavy proteinuria (+++), microscopic hematuria (+++). Arterial blood gas analysis indicated a mixed acid–base disorder, with metabolic acidosis accompanied by respiratory alkalosis, and mild hyponatremia (Na⁺ 134 mmol/L). Biochemistry showed severe azotemia (urea 24.87 mmol/L, creatinine 787.3 μmol/L), hypocalcemia (1.95 mmol/L), hyperuricemia (1077 μmol/L), hyperphosphatemia (2.72 mmol/L). Creatine kinase was moderately elevated (592 U/L; Table 1). Notably, liver function tests remained largely normal, with total bilirubin never exceeding the upper limit of normal (peak 18.9 μmol/L; reference 3.4–24.0). Renal ultrasound showed bilateral kidney enlargement, increased cortical echogenicity, and crystal deposition. The patient received empirical intravenous piperacillin–tazobactam (2.25 g every 8 hours) and nebulized corticosteroids. Due to persistent oliguria and worsening renal function, intermittent hemodialysis was initiated on hospital day 2.

**Table 1:** Laboratory data on admission.

Parameter	Recorded value	Standard value	Unit
White blood cell count	10.23	3.50–9.50	×10^9^/L
Neutrophil count	8.78	1.80–6.30	×10^9^/L
Hemoglobin	126	130–175	g/L
Platelet count	133	125–350	×10^9^/L
C-reactive protein	40.2	≤10.0	mg/L
Procalcitonin	0.52	≤0.25	ng/mL
International normalized ratio	1.26	0.85–1.20	
Activated partial thromboplastin time	26.6	21.6–32.4	s
Fibrinogen	3.08		
d-dimer	310	≤550.0	μg/L
Serum potassium	3.68	3.50–5.30	mmol/L
Serum sodium	133.6	137.0–147.0	mmol/L
Serum chloride	100.9	99.0–110.0	mmol/L
Serum calcium	1.95	2.10–2.80	mmol/L
Alanine aminotransferase	39	<50	U/L
Blood glucose	7.07	4.20–6.10	mmol/L
blood urea nitrogen	24.87	3.20–7.10	mmol/L
Creatinine	787.3	57.0–97.0	μmol/L
Estimated glomerular filtration rate	6.85		mL/(min 1.73 m^2^)
Creatine phosphokinase	592	55–170	μmol/L
Serum amylase	167	30–110	U/L
Osmolarity	318	275–300	mOsm/L
Urine protein	+ + +	(−)	
Urine microalbumin	+ + +	(−)	
Urine sediment red blood cell count	83.8	≤18	/μL
PH	7.352	7.350–7.450	
Partial pressure of arterial oxygen	102	80–100	mm Hg
Partial pressure of arterial carbon dioxide	29.5	35.0–45.0	mm Hg
Bicarbonate	15.9	22.0–27.0	mmol/L
Standard bicarbonate	18	21.3–24.8	mmol/L
Actual base excess	−8	−3.0 to +3.0	mmol/L
Standard base excess	−8.6	−3.0 to +3.0	mmol/L
Anion gap	15.1	8.0–16.0	mmol/L

**Figure 1.**
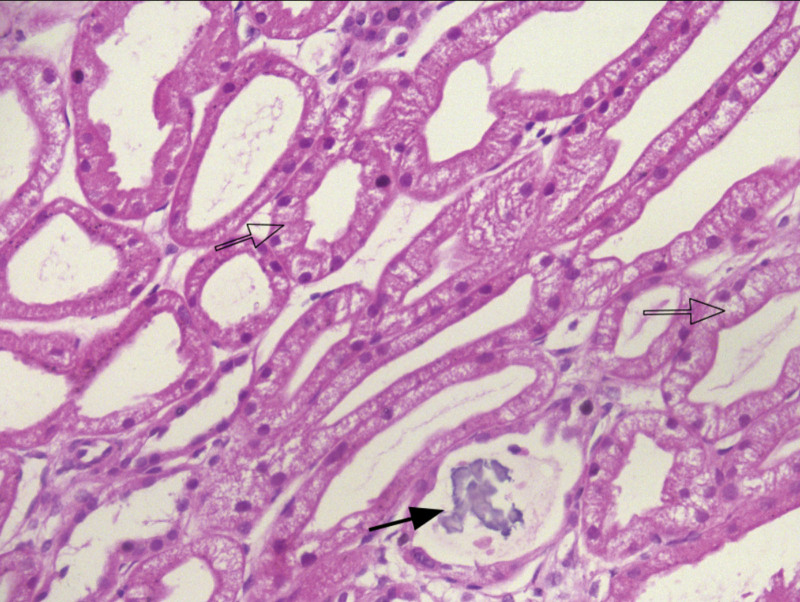
Renal biopsy findings (Hematoxylin and Eosin stain, original magnification ×400). Black arrows: Calcium salt deposits are observed within the tubular lumen. Open arrows: Marked vacuolar degeneration is present in the tubular epithelial cells.

**Figure 2.**
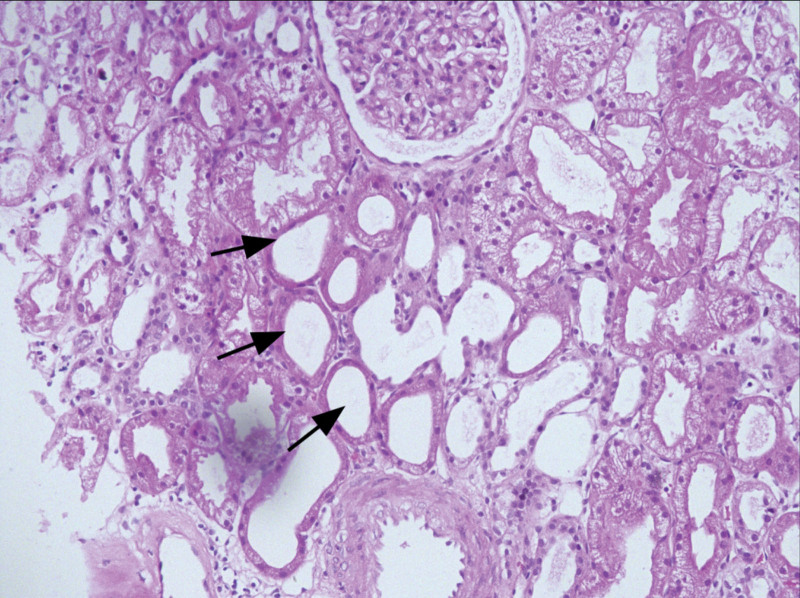
Renal biopsy findings (Hematoxylin and Eosin stain, original magnification ×200). Black arrows: multifocal loss of brush border and flattening of tubular epithelial cells.

**Figure 3.**
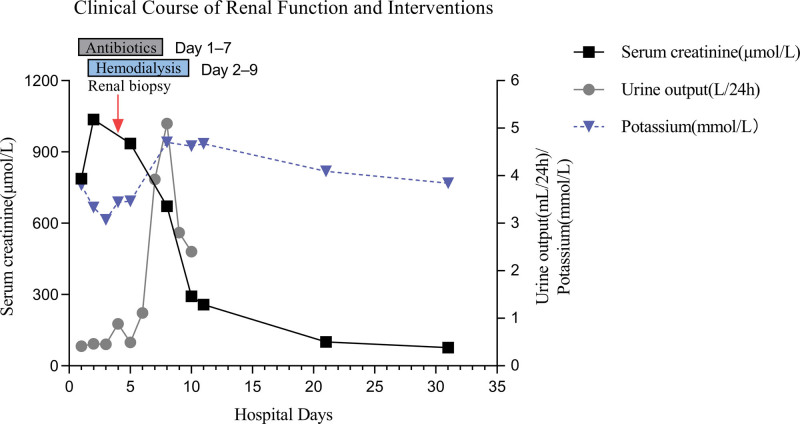
Clinical course of renal function and intervention.

A renal biopsy was performed on hospital day 4. Light microscopy confirmed the typical features of acute tubular injury (ATI; Figs. 1 and 2). These findings were supported by immunofluorescence and electron microscopy, which ruled out immune complex deposition and revealed ischemic endothelial vacuolization, respectively. The patient received 7 sessions of hemodialysis in total (Fig. 3). His urine output gradually improved, exceeding 1500 mL/d by hospital day 9. Serum creatinine declined to 292.0 μmol/L, and he was discharged on hospital day 11. At 1 month follow-up, renal function had completely normalized (serum creatinine 98 μmol/L), and the patient reported no residual symptoms.

The patient expressed initial anxiety about the diagnosis and dialysis. Clear explanations from the medical team alleviated his concerns and promoted cooperation. At follow-up, he was satisfied with his full recovery and emphasized the value of effective communication.

## 3. Discussion

AKI related to drowning is typically the result of multiple interrelated pathogenic mechanisms.^[6]^ In this case, ischemia-reperfusion injury was likely the predominant factor.^[7]^ During the drowning episode, the patient experienced prolonged and intense physical struggle, which may have redirected blood flow preferentially toward the skeletal muscles, resulting in transient renal hypoperfusion. Given the kidney’s high metabolic activity and oxygen demand – second only to the myocardium – it is particularly vulnerable to hypoxic conditions.^[8]^ Even brief ischemic episodes can be exacerbated by subsequent reperfusion, further amplifying renal injury.^[9]^

Following ischemia, the renal endothelium and parenchymal cells rapidly initiate a proinflammatory cascade, leading to a robust inflammatory response.^[10]^ Endothelial damage promotes the expression of adhesion molecules, resulting in leukocyte sequestration and microcirculatory dysfunction.^[10,11]^ These processes contribute to injury of tubular epithelial cells, impairing their reabsorptive capacity and leading to electrolyte disturbances and acid–base imbalance.

Renal biopsy in this patient revealed multifocal loss of brush border and vacuolar degeneration of tubular epithelial cells, histological features consistent with ATI, thereby supporting this pathophysiological mechanism. Although the clinical presentation was highly suggestive of ischemic ATI, a renal biopsy was performed to definitively confirm the diagnosis, exclude other potential pathologies, and evaluate the extent of tubular damage. The histological findings provided critical information for prognostic assessment and facilitated discussions with the patient and his family regarding the anticipated clinical course.

Renal biopsy-confirmed AKI following drowning remains an uncommon finding in medical literature. In a large series of 562 near-drowning patients, Gorelik et al identified hypoxic tubular injury as the most probable mechanism for AKI, though their study lacked histological confirmation.^[4]^ According to the comprehensive review by Ma et al of cases and series from 1996 to 2016, supplemented by recent biopsy-proven case reports, the predominant histopathological pattern in drowning-associated AKI consistently demonstrates ATI.^[12]^ Notably, the total number of biopsy-verified cases remains remarkably scarce, suggesting that histological documentation of this pathological mechanism continues to be rare. Our case aligns with these findings, showing biopsy-confirmed ATI without primary glomerular pathology. The renal injury in our patient was relatively mild, with complete functional recovery achieved within 1 month.

Rhabdomyolysis is another recognized cause of AKI following near-drowning, particularly in individuals who experience intense physical exertion or prolonged muscle hypoxia. Myoglobin released from injured skeletal muscle can cause direct tubular toxicity, promote cast formation, and induce intrarenal vasoconstriction, further compromising renal perfusion.^[13,14]^ However, in our patient, there was insufficient evidence to support rhabdomyolysis. On the day of admission – which was the third day after the drowning event – creatine kinase was only mildly elevated (592 U/L), myoglobin was not measured, and urine sediment showed no granular or pigment casts.

Volume depletion is a recognized contributor to AKI following near-drowning. Submersion can lead to intravascular volume depletion due to insensible fluid loss and third-spacing. When compounded by systemic vasodilation from inflammatory responses, renal perfusion may further decline. In this case, although physical exertion may have caused some fluid loss, no hypotension or clinical dehydration was observed on admission. Thus, hypoperfusion due to hypovolemia lacks definitive evidence here.

Drug-induced nephrotoxicity should also be taken into consideration. Pulmonary inflammation is common in drowning patients, and empirical antibiotic therapy is often required to prevent or treat infection – our patient was no exception. Piperacillin–tazobactam was initiated on the day of admission (2.25 g every 8 hours via microinfusion pump for a total of 7 days).

This antibiotic is primarily eliminated through the kidneys. Previous studies have suggested that piperacillin–tazobactam–associated AKI often presents as acute interstitial nephritis, and its risk appears to be closely related to early cumulative exposure.^[15]^ Therefore, given the continued rise in serum creatinine on day 2 of hospitalization, we could not rule out a potential aggravating effect of this agent on the preexisting kidney injury.

However, renal biopsy revealed only mild interstitial edema and scattered inflammatory cell infiltration, without the classic histological features of acute interstitial nephritis. We also considered other potential causes in the context of freshwater drowning. Specifically, leptospirosis was a diagnostic consideration. However, this possibility was deemed low given the absence of jaundice, consistently normal total bilirubin levels, and lack of typical symptoms such as conjunctival injection or myalgia throughout the hospitalization.

In this case, renal biopsy-confirmed ATI, supporting a multifactorial pathogenesis. Individualized hemodynamic management, early adjustment of medications, and timely initiation of renal replacement therapy were critical for renal recovery. This case underscores the critical role of renal biopsy in identifying the underlying cause of AKI when clinical manifestations are nonspecific or potentially multifactorial. Although the initial presentation suggested mechanisms such as ischemia-reperfusion injury and rhabdomyolysis, the biopsy-confirmed diagnosis of ATI provided definitive pathological evidence. The patient experienced a dialysis-dependent phase followed by full renal recovery, highlighting the value of integrating histopathology, therapeutic response, and individualized management. AKI following drowning is often the result of multiple concurrent insults, warranting continuous and comprehensive diagnostic reassessment throughout the disease course.

## 4. Conclusion

This case underscores that AKI is a salient complication of near-drowning, which can develop without preceding cardiopulmonary collapse. This observation mandates vigilance for AKI across the entire spectrum of drowning severity, rather than solely in cases of profound physiological compromise. Prompt recognition, individualized fluid and hemodynamic management, and appropriate use of renal biopsy when needed are crucial for guiding treatment and optimizing renal outcomes. This case adds to the limited number of biopsy-verified reports of drowning-associated AKI, underscoring the diagnostic and prognostic importance of renal biopsy in selected patients.

## Author contributions

**Writing – original draft:** Jihao Xu.

**Writing – review & editing:** Bin Zhu.

## References

[1] World Health Organization. Global Report on Drowning: Preventing a Leading Killer. World Health Organization; 2014. https://www.un.org/en/node/138359.

[2] Drowning: a silent killer [editorial]. Lancet. 2017;389:1859. 28513436 10.1016/S0140-6736(17)31269-2

[3] Bellomo R Kellum JA Ronco C. Acute kidney injury. Lancet (London, England). 2012;380:756–66. 22617274 10.1016/S0140-6736(11)61454-2

[4] Gorelik Y Darawshi S Yaseen H Abassi Z Heyman SN Khamaisi M. Acute renal failure following near-drowning. Kid Int Rep. 2018;3:833–40. 10.1016/j.ekir.2018.02.007 PMC6035158 29989059

[5] Heyman SN Gorelik Y Zorbavel D. Near-drowning: new perspectives for human hypoxic acute kidney injury. Nephrol Dial Transplant. 2020;35:206–12. 30768198 10.1093/ndt/gfz016

[6] Seong EY Rhee H Lee N. A case of severe acute kidney injury by near-drowning. J Korean Med Sci. 2012;27:218–20. 22323873 10.3346/jkms.2012.27.2.218 PMC3271299

[7] Enescu DM Parasca SV Badoiu SC. Hypoxia-inducible factors and burn-associated acute kidney injury—a new paradigm? Int J Mol Sci . 2022;23:2470. 35269613 10.3390/ijms23052470 PMC8910144

[8] Zhang X Agborbesong E Li X. The role of mitochondria in acute kidney injury and chronic kidney disease and its therapeutic potential. Int J Mol Sci . 2021;22:11253. 34681922 10.3390/ijms222011253 PMC8537003

[9] Nørgård MO Svenningsen P. Acute kidney injury by ischemia/reperfusion and extracellular vesicles. Int J Mol Sci . 2023;24:15312. 37894994 10.3390/ijms242015312 PMC10607034

[10] Bonventre JV Zuk A. Ischemic acute renal failure: an inflammatory disease? Kidney Int. 2004;66:480–5. 15253693 10.1111/j.1523-1755.2004.761_2.x

[11] Willinger CC Schramek H Pfaller K Pfaller W. Tissue distribution of Neutrophils in postischemic acute renal failure. Virchows Archiv B Cell Pathol Include Mol Pathol. 1992;62:237–43. 10.1007/BF02899687 1359696

[12] Ma TKW Chow KM Leung CB Szeto CC Li PKT. Near-drowning related acute kidney injury. Clin Nephrol. 2016;85:305–8. 27007870 10.5414/CN108840

[13] Lu Y Neyra JA. How I treat rhabdomyolysis-induced AKI? Clin J Am Soc Nephrol. 2024;19:385–7. 37934632 10.2215/CJN.0000000000000372 PMC10937018

[14] Bosch X Poch E Grau JM. Rhabdomyolysis and acute kidney injury. N Engl J Med. 2009;361:62–72. 19571284 10.1056/NEJMra0801327

[15] Tang Girdwood S Hasson D Caldwell JT. Relationship between piperacillin concentrations, clinical factors and piperacillin/tazobactam-associated acute kidney injury. J Antimicrob Chemother. 2023;78:478–87. 36545869 10.1093/jac/dkac416 PMC10169424

